# Constructing interface engineering and tailoring a nanoflower-like FeP/CoP heterostructure for enhanced oxygen evolution reaction†

**DOI:** 10.1039/d3ra01096a

**Published:** 2023-05-16

**Authors:** Linhua Wang, Hua Yang, Lulan Wang, Yunwu Li, Wenning Yang, Xu Sun, Lingfeng Gao, Mingyu Dou, Dacheng Li, Jianmin Dou

**Affiliations:** a Shandong Provincial Key Laboratory of Chemical Energy Storage and Novel Cell Technology, School of Chemistry and Chemical Engineering, Liaocheng University 252059 Liaocheng P. R. China; b Key Laboratory of Interfacial Reaction & Sensing Analysis in Universities of Shandong, School of Chemistry and Chemical Engineering, University of Jinan Jinan 250022 Shandong P. R. China

## Abstract

The inexpensive and highly efficient electrocatalysts toward oxygen evolution reaction (OER) in water splitting electrolysis have displayed promising practical applications to relieve energy crisis. Herein, we prepared a high-yield and structurally regulated bimetallic cobalt-iron phosphide electrocatalyst by a facile one-pot hydrothermal reaction and subsequent low-temperature phosphating treatment. The tailoring of nanoscale morphology was achieved by varying the input ratio and phosphating temperature. Thus, an optimized FeP/CoP-1-350 sample with the ultra-thin nanosheets assembled into a nanoflower-like structure was obtained. FeP/CoP-1-350 heterostructure displayed remarkable activity toward the OER with a low overpotential of 276 mV at a current density of 10 mA cm^−2^, and a low Tafel slope of only 37.71 mV dec^−1^. Long-lasting durability and stability were maintained with the current with almost no obvious fluctuation. The enhanced OER activity was attributed to the presence of copious active sites from the ultra-thin nanosheets, the interface between CoP and FeP components, and the synergistic effect of Fe–Co elements in the FeP/CoP heterostructure. This study provides a feasible strategy to fabricate highly efficient and cost-effective bimetallic phosphide electrocatalysts.

## Abstract 2

A nanoflower-like bimetallic FeP/CoP-1-350 heterostructure electrocatalyst with rich interfaces shows excellent OER electrocatalytic activity.

## Graphical abstract

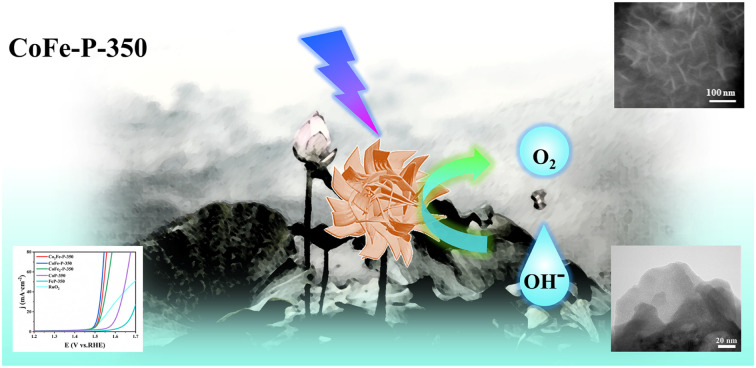

## 1 Introduction

Hydrogen energy, the future renewable and clean energy, is considered an ideal alternative to address the current depletion of traditional energy and excessive carbon emissions.^1–3^ So far, the electrolysis of water is regarded as an effective energy conversion technology to obtain hydrogen.^4,5^ However, the oxygen evolution reaction (OER), one of the two half-reactions of water cracking, with the inherent sluggish kinetics and high overpotential, limits the industrial application of monolithic catalysis in water splitting electrolysis.^6,7^ It stimulates the researchers to explore high-efficient electrocatalysts to accomplish the water splitting. The Ir- and Ru-based noble metal catalysts are the benchmarks for OER and demonstrate superior electrocatalytic activity.^8–10^ However, the scarcity and high price of such materials hinder their large-scale application in the commercial production of hydrogen. Therefore, the synthesis of low-cost, highly efficient, and durable OER electrocatalysts is a huge challenge and needs to be addressed urgently.

Generally, a low-cost electrocatalyst with superior OER activity is desirable for practical applications. Recently, considerable efforts have been focused on the development of the Earth's abundance and inexpensive non-precious element-based catalysts.^11,12^ Selenides,^13,14^ phosphides,^15,16^ nitrides,^17,18^ oxides,^19,20^ carbides,^21,22^ sulfides,^23,24^ and hydroxides^25,26^ have been applied as electrocatalysts toward OER. Among them, transition metal phosphides (TMPs) have exhibited considerable advantages, such as earth-abundance, cost-effectiveness, approximately spherical triangular prism unit structure, indicating more active sites, high conductivity, and long sustainability in alkaline solutions.^27,28^ CoP-based catalysts with strong redox activity and high corrosion resistance are promising candidates in water splitting electrolysis and have showcased excellent catalytic activity due to their durability in reaction.^29–31^ However, in order to obtain the desired electrocatalytic activity and achieve the practical application, CoP materials still need to be considered for enhancing the relatively poor electrical conductivity and increasing accessible active sites. Currently, nanostructuring and tuning electronic structures have been proposed to improve catalytic performance.^32,33^ Many efforts revealed that tailoring the nanoscale morphology is very significant in the synthetic process of electrocatalysts to optimize the electrocatalytic activity and enhance electrochemical performances by increasing the exposure of more active sites, specific surface area, and shortening charge transfer distances.^34–36^ Guo *et al.* found that the microstructure and morphology of CoG nanospheres could be tuned by varying the water content, and the resulting hollow CoG nanospheres constructed through the assembly of ultra-thin nanosheet displayed high specific area and supplied more active sites.^37^ Wang *et al.* fabricated a hollow nano-frame Co–Fe–P bimetallic catalyst by increasing the aging temperature, which achieved remarkable catalytic performances toward OER.^38^ Furthermore, by elemental doping or composite tuning, the electronic structure can form the clarifying interface to facilitate the charge transfer in interface engineering to expose copious active sites.^4,38,39^ Due to the wide distribution, low-cost, and multiple chemical states of Fe element, the construction of bimetallic Fe–Co–P materials will achieve higher OER catalytic activity and promisingly reserve in the industry with the help of the desirable electrocatalytic performance. Although the combination of Fe–Co elements in phosphatized products has captured the attention of researchers, only a few studies have focused on electrocatalytic OER.^40^ A hollow prisms Fe-doped CoP exhibited abundant mesoporous structure and extraordinary OER performance in 1.0 M KOH.^41^ Xu *et al.* synthesized a two-dimensional mesh-like porous Fe-doped CoP nanosheet, which displayed excellent mass transfer kinetics and a multitude of active sites.^42^ However, the multi-step synthetic steps such as Lewis acid exchange in the synthesis of precursors, and low yield of precursor led to the wasting of resources and diseconomy.^29,43–45^ Therefore, it is necessary to simplify the synthetic process and efficiently prepare high-yield catalysts with excellent activity to meet the future, large-scale practice applications.

In view of the above considerations, an ultra-thin nanosheet assembled into a nanoflower-like structure catalyst (called FeP/CoP-1-350) was prepared by a facile one-pot hydrothermal reaction and subsequent low-temperature phosphating of the precursor. We investigated the effect of the ratio of starting materials and phosphating temperature on the catalyst morphology and OER performance. Under optimized conditions, FeP/CoP-1-350 electrocatalyst displayed a considerable number of ultra-thin nanosheets with even distribution of each element and revealed superior OER electrocatalytic activity as a result of the synergistic effect between Co and Fe elements and the interface between CoP and FeP components in FeP/CoP heterostructure. This work provided a fresh approach for preparing TMP electrocatalysts with a high yield and superior activity toward OER by tuning the composition and morphology.

## 2 Experimental section

### 2.1. Materials

Cobalt nitrate hexahydrate (Co(NO_3_)_2_·6H_2_O) and sodium hypophosphite (NaH_2_PO_2_·H_2_O) were supplied by Aladdin. Iron nitrate nonahydrate (Fe(NO_3_)_3_·9H_2_O) and hexamethylenetetramine (C_6_H_12_N_4_) were bought from Weng Jiang Reagent and Tianjin Fuchen Chemical Reagents Factory, respectively. All chemicals were analytical grade and used without further purification.

### 2.2. Synthesis of electrocatalysts

#### 2.2.1 Synthesis of Co–Fe precursors

Firstly, 10 mmol C_6_H_12_N_4_ and 4 mmol of admixture of metal nitrate in different mole ratios of Fe/Co (1/2, 1, 2/1) were dissolved in 60 ml of ethanol. After the magnetic stirring for 24 h, the above-mixed solution was sealed in a 100 ml Teflon-lined autoclave and heated at 100 °C for 12 h, then, it was naturally cooled to room temperature. The precipitated samples were centrifugated, washed with ethanol three times, and dried at 60 °C for 12 h in an oven. Finally, the collected samples were denoted as Co_2_Fe pre, CoFe pre, and CoFe_2_ pre, respectively. The preparation processes of Co pre and Fe pre were similar to the syntheses of Co–Fe precursors, except that Co(NO_3_)_2_·6H_2_O and Fe(NO_3_)_3_·9H_2_O were exclusively adopted.

#### 2.2.2 Synthesis of Co–Fe–P samples

Typically, 100 mg of the above Co–Fe precursors and 1.0 g of NaH_2_PO_2_·H_2_O were placed in two porcelain boats with NaH_2_PO_2_·H_2_O on the upstream side, which was located in the center of a quartz tube in a tubular furnace, and then it was heated to 350 °C in N_2_ atmosphere at a rate of 2 °C min^−1^ and treated for 2 h at 350 °C. The as-synthesized products were expressed as FeP/CoP-0.5-350, FeP/CoP-1-350, and FeP/CoP-2-350, respectively. The fabricated processes of CoP-350, and FeP-350 were similar to those of Co–Fe–P samples. The Co–Fe–P samples were further investigated by changing the phosphating temperature in the range of 300 to 500 °C, and the resulting products were named FeP/CoP-1-300, FeP/CoP-1-400, and FeP/CoP-1-500, respectively.

### 2.3. Characterizations and measurements

The crystal phases of all the catalysts were confirmed by X-ray diffraction (XRD) on a Smart Lab 9 diffractometer (Japan). The chemical composition and valence state of surface elements were examined by X-ray photoelectron spectroscopy (XPS) measurements on a Thermo ESCALAB Xi^+^ instrument (USA). A scanning electron microscope (SEM, Thermo Fisher Science FIB-SEM GX4 (USA)), transmission electron microscope (TEM), and high-resolution transmission electron microscope (HRTEM, Talos F200X G2 (USA)) were used to investigate the morphologies and microstructure of all samples.

### 2.4. Electrochemical measurements

Electrochemical measurements were carried out in 1 M KOH solution using a Gamry Reference 3000 electrochemical workstation. A typical three-electrode system was adopted, including a catalyst-modified glassy carbon electrode as the working electrode (glassy carbon electrode (GCE), diameter 5 mm), Hg/HgO electrode as the reference electrode, and carbon rod as the counter electrode. The modification method of the working electrode was as follows: 3.2 mg catalyst sample, 1.6 mg carbon powder (Cabot Vulcan XC-72), and 20 μl 5 wt% Nafion ethanol solution were added into the mixed solution (containing 340 μl water and 140 μl ethanol), and the ultrasonic treatment was carried out for 30 min; then, 10 μl of the catalyst ink was dropped on the polished GCE and dried naturally in the air. According to the Nernst equation relative, all the reported potentials are converted to the reversible hydrogen electrode (RHE): *E*_RHE_ = *E*_Hg/HgO_ + 0.0592 pH + 0.0977. The polarization curve was obtained by linear sweep voltammetry (LSV) at the scanning rate of 5 mV s^−1^. All potentials were referenced by *iR*-compensated reversible hydrogen electrode (RHE). Electrochemical impedance spectroscopy (EIS) measurements were performed with AC voltage 10 mV at the frequency from 100 kHz to 0.01 Hz, and the potential difference (*R*_s_) of the solution resistance was deduced. Electrochemical double-layer capacitance (*C*_dl_) was tested at different scanning rates (*v* = 20, 40, 60, 80, and 100 mV s^−1^) in the non-Faraday region, which was used to evaluate the electrochemically active surface area (ECSA). In order to obtain the stability and durability of the sample, we carried out 1000 CV cycles and *i*–*t* measurements.

## 3 Results and discussion

### 3.1. Characterization of structure, morphology, and composition of the catalysts

**Scheme 1:**
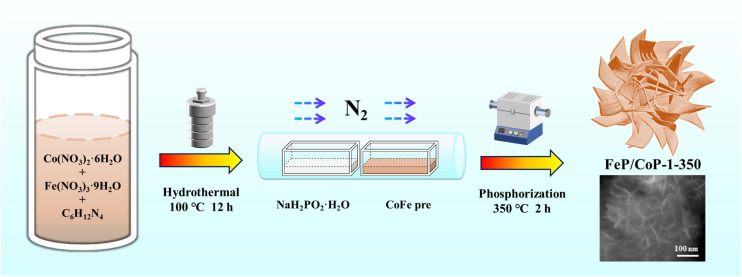
Schematic diagram of the synthetic process of FeP/CoP-1-350.

The optimal conditions of the synthetic process of FeP/CoP-1-350 are described in Scheme 1. All samples were synthesized following a similar strategy. The structure, morphology, as well as composition of all samples, are discussed and compared in detail.

**Fig. 1:**
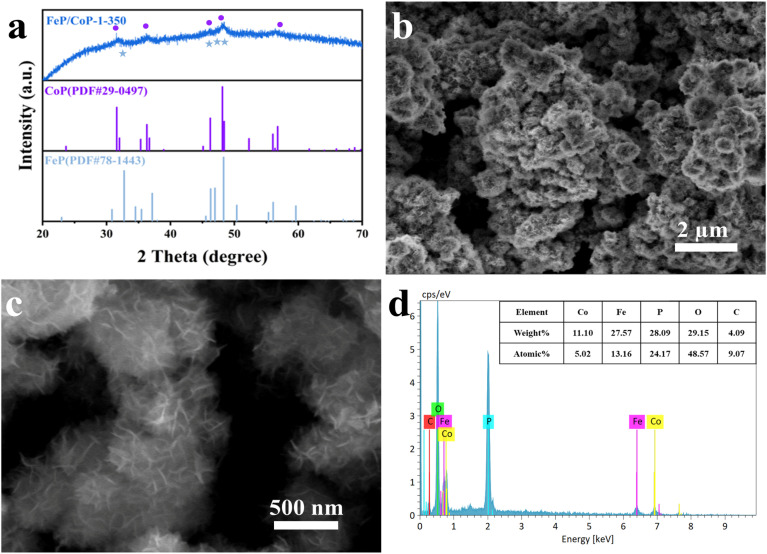
(a) XRD patterns; (b and c) SEM images; (d) EDS spectrum of FeP/CoP-1-350.

As shown in Fig. 1a, the crystal structures of the catalysts can be observed by XRD. The diffraction peaks at 31.6°, 36.3°, 46.2°, 48.1°, and 56.8°, respectively, correspond to the (011), (111), (112), (211), and (301) crystal planes of CoP (PDF # 29-0497); the characteristic peaks at 32.7°, 46.3°, 47.0°, and 48.3° are respectively attributed to the (011), (112), (202) and (211) crystal planes of FeP (PDF # 78-1443), indicating that CoP and FeP coexist in FeP/CoP-1-350 sample.^39^ Although the input ratio in samples was different, the position of the diffraction peaks was consistent (Fig. S1b†). As the phosphating temperature increased, the full-width at half-maximum (FWHM) of the diffraction peaks in the XRD pattern gradually became more obvious and narrower (Fig. S1c†), suggesting the enhancement of the crystallinity of phosphatized products.^46^ Although the phosphating temperature ranged from 300 to 500 °C, considerable reports also proved that the phosphide phases could be successfully achieved and kept stable at the high phosphating treatment, as shown in their XRD patterns.^40,47–50^ So this also showed that the phosphatized products discussed here could be successfully constructed.

The surface morphology and microstructure of FeP/CoP-1-350 were investigated by SEM and TEM. The SEM image displays nanoflower-like morphology features (Fig. 1a and b), while the enlarged SEM image exhibits the ultra-thin nanosheets with lamellar stacking structure. The comparison of the morphology of FeP/CoP-0.5-350 and FeP/CoP-2-350 samples prepared using different proportions of raw materials showed that the former had irregularly distributed ultra-thin nanosheets, while the latter demonstrates a very small amount of ultra-thin nanosheets and amorphous nanoparticles (Fig. S2a and b†). As a result, the input ratio of raw materials has a substantial influence on the morphology of the samples. When the input molar ratio of Co and Fe elements was 1 : 1, the perfect nanoflower structure containing ultra-thin nanosheets appeared. At the optimal molar ratio, the phosphating temperature also gave rise to the change in the morphology of the resulting samples. As shown in Fig. S2e–g,† as the phosphating temperature increased from 300 to 500 °C, the CoFe–P samples first displayed a handful of ultra-thin nanosheets, then perfect nanoflower-like structure composed of ultra-thin nanosheets, lastly a large number of nanoparticles melted or combined together. Therefore, we can infer that the ultra-thin nanosheets began to form at the phosphating temperature of 300 °C, and then the perfect nanoflower-like structure with ultra-thin nanosheets appeared at 350 °C, as the phosphating temperature further increased, the ultra-thin nanosheets began to twist at 400 °C and lastly melted into nanoparticles at 500 °C. Fig. S2c and d† show the morphology of the CoP-350 sample with irregular nanosheets and FeP-350 with irregular nanoparticles accompanying a small amount of nanosheets on the surface of the nanoparticles. As such, at the appropriate molar ratio of the starting materials (Fe/Co = 1 : 1) and phosphating temperature (350 °C), the resulting product demonstrated the perfect nanoflower-like structure, which would promote more active site exposure and enhance the OER activity.

Furthermore, the elemental composition of FeP/CoP-1-350 was quantitatively analyzed from the energy-dispersive X-ray spectroscopy (EDS) spectrum, as shown in Fig. 1d. The atomic ratio of Co/Fe/P in FeP/CoP-1-350 sample was about 1 : 2.62 : 4.81, C and O elements were derived from the decomposition of hexamethylenetetramine and the superficial oxidation of FeP/CoP-1-350, respectively, because of the exposure to air.^51^ The existence of the C element accelerates the charge transfer and further enhances OER performance. Moreover, EDS spectra and the elemental compositions from quantitative analysis of different samples with various starting materials ratios or phosphating temperatures are given in Fig. S3.† The analysis of the EDS spectra found that the oxygen content in the sample increased, which suggested that the interaction of Co and Fe elements intensified the oxidation of the sample.^52^ As the phosphating temperature increased from 300 °C to 500 °C, Fe content in the sample first decreased, then increased. A similar change was also found in P content in other reported phosphatized products.^36^ This may be related to the morphology of samples. Meanwhile, it further indicates that the phosphating temperature can tune the composition of elements and morphology to form optimal electrocatalysts toward excellent catalytic performance.

**Fig. 2:**
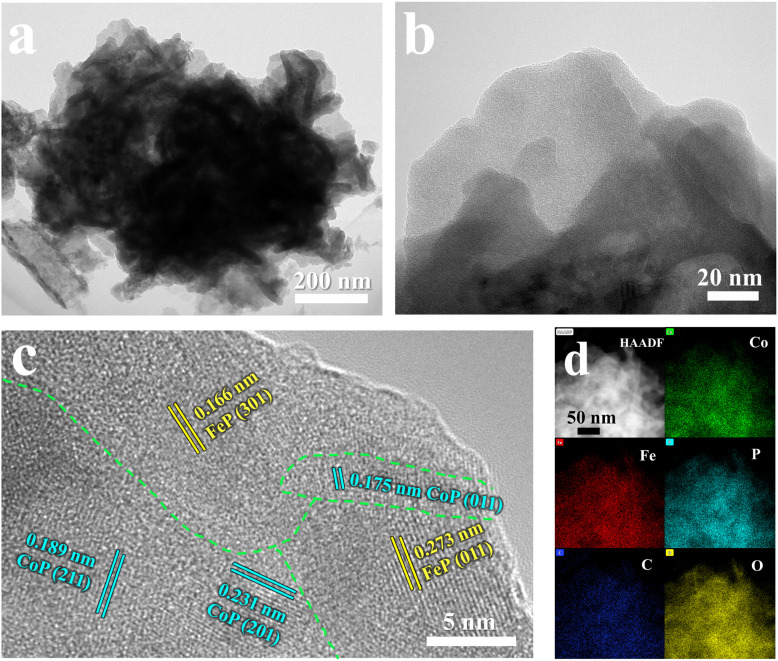
(a and b) TEM images; (c) HRTEM images; (d) HAADF-STEM image and the corresponding elemental mapping images of Co, Fe, P, C, and O of FeP/CoP-1-350.

The nanoflower-like structure of the FeP/CoP-1-350 sample formed by the stacking of ultra-thin nanosheets was further demonstrated from TEM images (Fig. 2a and b). The crystal structure of the ultra-thin nanosheets of the FeP/CoP-1-350 sample was analyzed using the high-resolution TEM (HRTEM) (Fig. 2c). Impressively, two lattice fringes with a spacing of 0.166 nm and 0.273 nm are attributed to the (301) and (011) phase of FeP, respectively, while the other three lattice fringes with spacings of 0.175 nm, 0.189 nm, and 0.231 nm correspond to the (011), (211) and (201) phase of CoP, respectively, indicating the co-existence of FeP and CoP. There is an obvious interface or joint between FeP and CoP, which illustrates the synergistic effect between the two composites. These results confirm that the FeP/CoP-1-350 sample is composed of a homogeneous mixture phase of CoP and FeP, which are closely contacted to create a unique CoP/FeP interface. The high-angle annular dark-field scanning TEM (HAADF-STEM) and the corresponding elemental mapping images show the even distribution of Co, Fe, P, C, and O elements in the FeP/CoP-1-350 sample (Fig. 2d), further suggesting CoP and FeP are closely mixed.

**Fig. 3:**
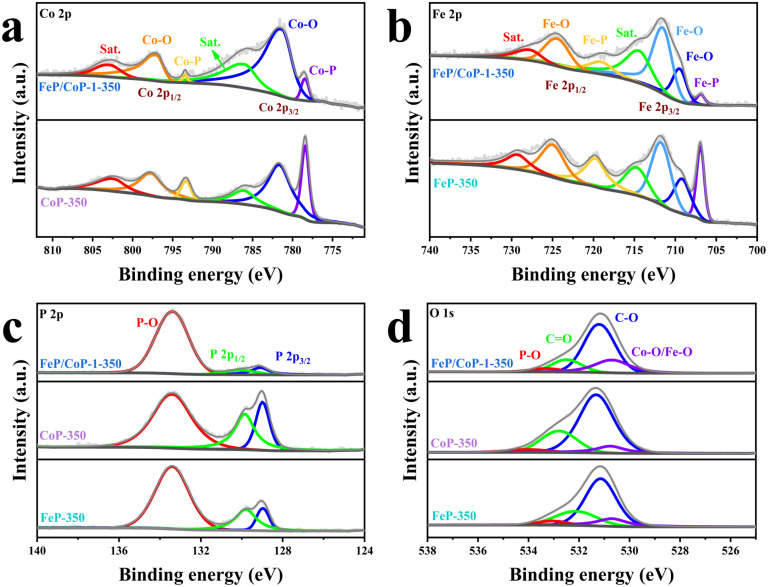
High-resolution XPS spectra for (a) Co 2p; (b) Fe 2p; (c) P 2p; (d) O 1s of FeP/CoP-1-350, CoP-350, and FeP-350.

The surface chemical states of the samples were investigated using XPS, and the full spectra of XPS reveal the existence of Fe, Co, P, C, and O elements (Fig. S4a†), where the binding energy at ∼710 eV demonstrates the different peak shapes observed for FeP/CoP-1-350 and CoP-350 samples (Fig. S4a†), indicating the existence of Fe element in FeP/CoP material. As shown in Fig. 3a, the high-resolution XPS spectra of Co 2p exhibited two peaks around 778.5 eV and 793.4 eV from Co^3+^, corresponding to Co 2p_3/2_ and 2p_1/2_, respectively, the characteristic peaks of Co–P. In comparison with the Co metal peak (777.9 eV), a slight positive shift was observed in the binding energy of Co 2p_3/2_ in Co–P, indicating a slight electron density transfer from Co to P.^53^ The peaks at 781.6 eV and 797.3 eV are assigned to the spin-orbitals of Co 2p_3/2_ and 2p_1/2_, respectively, pointing to Co species in Co–O. Two satellite peaks at 786.5 eV and 803.3 eV are attributed to the shake-up excitation of Co^2+^.^54,55^ In the high-resolution XPS spectra of Fe 2p (Fig. 3b), the peaks at 706.9 eV and 719.1 eV from Fe^*δ*+^ belong to the spin-orbitals of Fe 2p_3/2_ and 2p_1/2_ in Fe–P, respectively; the peaks are attributed to Fe–O bonds located at 709.5 (2p_3/2_), 711.5 (2p_3/2_), and 724.4 (2p_1/2_), respectively.^56,57^ The satellite peaks of Fe 2p are located at 714.4 eV and 727.8 eV.^40,50^ The comparison of the M–O peak area shows that Co–O and Fe–O species occupy a larger percentage in FeP/CoP-1-350 than that in CoP-350 and FeP-350. This indicates the interaction between Co and Fe elements intensifies the oxidation degree of the sample surface.^52^ In other words, O element may originate from partially superficial oxidation, which facilitates enhancing the OER activity.^58^ The increase of Co–O and Fe–O peak area was also verified though P 2p spectra (Fig. 3c). The characteristic peaks at 129.1 eV and 130.1 eV correspond to P 2p_3/2_ and P 2p_1/2_ of P–M, respectively, indicating the formation of metal phosphides, while the peak at 133.4 eV is assigned to P–O, oxidized phosphide species owing to air exposure.^59^ For P 2p_3/2_, the binding energy (129.1 eV) had a slight negative shift compared with the elemental P (130.0 eV), suggesting a slight electronic transfer occurring from Co to P.^60^ In O 1s spectra (Fig. 3d), four peaks around 530.7 eV, 531.2 eV, 532.5 eV, and 533.3 eV belong to Fe–O/Co–O, C–O, C=O, and P–O species, respectively.^49,61,62^ The characteristic peaks located at 284.4 eV, 286.0 eV, and 288.5 eV in C 1s spectra correspond to C–C, C–O, and O–C=O (Fig. S4b†), respectively.^63^

### 3.2. Electrochemical performances

**Fig. 4:**
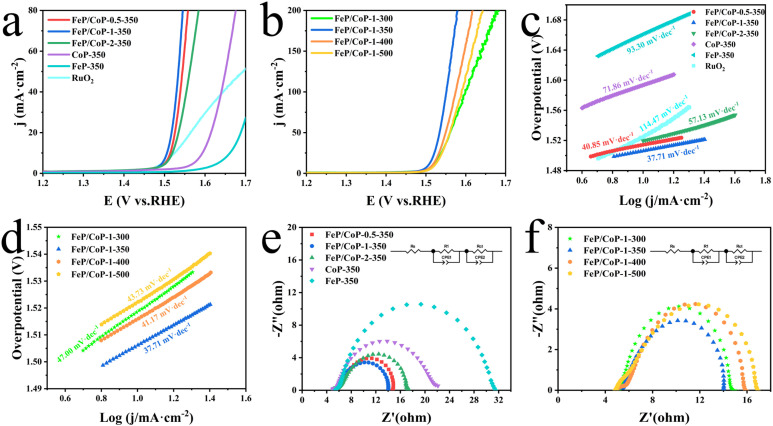
Electrochemical performance of all samples with various input ratios and phosphating temperatures, and RuO_2_. (a and b) LSV curves; (c and d) Tafel plots; (e and f) Nyquist plots and equivalent circuit.

The OER activities of the as-synthesized series of samples (FeP/CoP-1-350, FeP/CoP-1-300, FeP/CoP-1-400, FeP/CoP-1-500, CoP-350, FeP-350) and commercial RuO_2_ were investigated by LSV in 1 M KOH solution. As is known, the catalysts were usually evaluated using the overpotential at 10 mA cm^−2^. The lower overpotential indicates better catalytic activity.^64^ As shown in Fig. 4a, the LSV polarization curves revealed that FeP/CoP-1-350 achieved a current density of 10 mA cm^−2^ at an overpotential of only 276 mV, which is obviously lower than that of other samples (FeP/CoP-0.5-350, 284 mV; FeP/CoP-2-350, 290 mV; CoP-350, 364 mV; FeP-350, 432 mV), and commercial RuO_2_ (294 mV), suggesting that FeP/CoP-1-350 possesses superior OER activity. Such overpotential is comparable with the reported Co_0.8_Fe_0.2_P electrocatalyst.^52^ As the function of the proportion of Co/Fe, an optimal input ratio of Co/Fe obtained from the test results was 1 : 1. The in-depth study found that the phosphating temperature also affected the electrocatalytic efficiency. When the phosphating temperature changed from 300 to 500 °C, the overpotential varied following FeP/CoP-1-350 (276 mV) < FeP/CoP-1-400 (286 mV) < FeP/CoP-1-300 (289 mV) < FeP/CoP-1-500 (292 mV) (Fig. 4b). We carried out the comparison of the CoP/FeP sample, standard catalysts, and blank electrode, the experimental result showed that both standard catalysts and bare electrode displayed poor electrocatalytic activity (Fig. S5a†). So, we reached the following conclusion: the proportion of raw materials and the phosphating temperature influenced the electrocatalytic activity, and the electrocatalyst exhibited excellent OER performance under the optimal condition of the Fe/Co input ratio as 1 : 1 and the phosphating temperature at 350 °C.

Tafel slope is usually utilized to evaluate the OER kinetics, and the values can be derived from LSV curves. Comparison of the Tafel slopes (Fig. 4c and d) of all samples (FeP/CoP-1-350 (37.71 mV dec^−1^), FeP/CoP-0.5-350 (40.85 mV dec^−1^), FeP/CoP-2-350 (57.13 mV dec^−1^), CoP-350 (71.86 mV dec^−1^), FeP-350 (93.30 mV dec^−1^), FeP/CoP-1-300 (47.00 mV dec^−1^), FeP/CoP-1-400 (41.17 mV dec^−1^), FeP/CoP-1-500 (43.73 mV dec^−1^)) and RuO_2_ (114.47 mV dec^−1^), found that FeP/CoP-1-350 exhibited the lowest Tafel slope, suggesting OER exhibited considerably faster reaction kinetics.

The electrode catalytic kinetics and interfacial properties are also key parameters to evaluate OER electrocatalysts. Nyquist plots are shown in Fig. 4e and f, which exhibit a typical two-time-constant behavior.^65^ The fitting of Nyquist plots using a simplified equivalent electrical circuit gave the charge-transfer resistance (*R*_ct_) and uncompensated series resistance *R*_s_ (Table S1†). The smallest *R*_ct_ (8.86 Ω) indicated that FeP/CoP-1-350 has excellent conductivity and superior charge transfer efficiency.

**Fig. 5:**
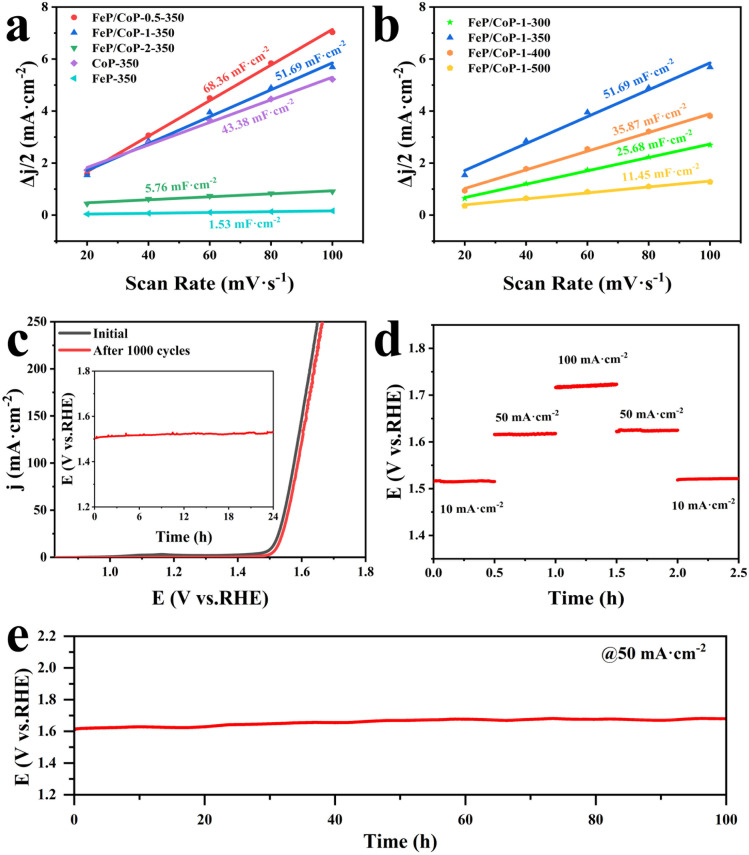
Electrochemical performance of samples with different input ratios and different phosphating temperatures. (a and b) The plots of current density *versus* scan rate; (c) LSV curves of the FeP/CoP-1-350 before and after 1000 cycles (inset: the chronopotentiometric curve of the FeP/CoP-1-350 at 10 mA cm^−2^); (d) multiple-step chronopotentiometric curve of FeP/CoP-1-350; (e) the chronopotentiometric curve of the FeP/CoP-1-350 at 50 mA cm^−2^.

The electrochemical active area (ECSA) was estimated through the measurement of double-layer capacitance (*C*_dl_) using CV. As presented in Fig. 5a, the *C*_dl_ value of FeP/CoP-1-350 was 51.69 mF cm^−2^, higher than that of CoP-350 (43.38 mF cm^−2^), FeP-350 (1.53 mF cm^−2^) and FeP/CoP-2-350 (5.76 mF cm^−2^), but lower than that of FeP/CoP-0.5-350 (68.36 mF cm^−2^). Although FeP/CoP-1-350 showed a lower *C*_dl_ value than FeP/CoP-0.5-350, it displayed a higher OER electrochemical activity. This abnormal phenomenon may have been derived from the synergistic action of bimetal and the change of electron density around Co and P centers because of the introduction of Fe regulating the electronic structure of the catalyst.^66–68^ The influence of phosphating temperature on ECSA is also discussed and the corresponding *C*_dl_ values are shown in Fig. 5b. The *C*_dl_ value of FeP/CoP-1-350 is higher than that of other materials obtained at different phosphating temperatures, indicating that too low or too high phosphating temperatures also influence OER activity. Usually, the *C*_dl_ is used to calculate the ECSA using the formula ECSA = *C*_dl_/40 μF cm^−2^,^65,69^ so the corresponding ECSA and normalized LSV curves are given in Table S1 and Fig. S7.†

The chemical stability and durability of samples are investigated to explore their practical applications. As can be seen from Fig. 5c, the polarization curve shows that the potential slightly shifts to the right after the continuous 1000 CV cycles, indicating that FeP/CoP-1-350 has excellent cyclic stability. The chronopotentiometry test measurement was carried out at an applied current density of 10 mA cm^−2^, as depicted in Fig. 5c (inset), the potential nearly remained constant without obvious fluctuation. Furthermore, the multiple-step chronopotentiometry curve of FeP/CoP-1-350 is shown in Fig. 5d, where each stage stayed in a steady state at different current densities, displaying excellent potential recoverability. In order to further evaluate the stability of the catalyst, chronopotentiometry measurement was carried out at 50 mA cm^−2^ current density. At first, the FeP/CoP-1-350 catalyst coating was wrapped on the GCE peels off after a 6 h test at high current densities. Later, the catalyst was transferred to carbon cloth (CC, 1 cm × 1 cm) according to the previous report, and the loading per unit area of CC was the same as on GCE.^15^ The potential did not display significant fluctuations after electrolysis at 50 mA cm^−2^ current density for 100 h (Fig. 5e), indicating that FeP/CoP-1-350 has satisfactory durability. Besides, the LSV curves of FeP/CoP-1-350/CC and bare CC are also shown in Fig. S5b.† As a result, the excellent cyclic stability and durability of FeP/CoP-1-350 proved that the electrocatalyst possibly has potential application in the future.

## 4 Conclusions

In summary, a series of bimetallic phosphide electrocatalyst FeP/CoP heterostructure with high yield was constructed through the simple, one-pot hydrothermal reaction and the subsequent phosphating reaction. The assembling of ultra-thin nanosheets into nanoflower-like structures can be designed and tailed by varying the ratios of starting materials and phosphating temperature. A FeP/CoP-1-350 electrocatalyst was fabricated under the optimal input ratio of Fe/Co (1 : 1) and phosphating temperature (350 °C) and achieved the tuning of morphology by nanostructuring. The ultra-thin nanosheet subunits and interfaces between CoP and FeP components provided plenty of active sites and high electron transfer possibility for OER reaction. Therefore, FeP/CoP-1-350 revealed the lowest overpotential (276 mV), the smallest Tafel slope (37.71 mV dec^−1^), and favorable stability at 1.0 M KOH. The outstanding electrocatalytic activity of this sample was ascribed to the synergistic effect of Co and Fe elements and the formation of ultra-thin nanosheets, which improved the OER kinetics and electrical conductivity. The facile and easy approach reported in this paper provided a high yield and inexpensive bimetallic phosphide electrocatalysts with superior OER performance in alkaline electrolytes.

## Author contributions

Linhua Wang: investigation, methodology, data curation, writing – original draft. Hua Yang: formal analysis, conceptualization, writing-reviewing and editing, funding acquisition. Lulan Wang: software, investigation. Yunwu Li: software. Wenning Yang: supervision. Xu Sun: validation. Lingfeng Gao: supervision. Mingyu Dou: methodology. Dacheng Li: supervision. Jianmin Dou: project administration, funding acquisition.

## Conflicts of interest

There are no conflicts to declare.

## Supplementary Material

RA-013-D3RA01096A-s001
