# Supplementary material for: Constructing interface engineering and tailoring a nanoflower-like FeP/CoP heterostructure for enhanced oxygen evolution reaction
Source: RSC Adv. 2023 May 16;13(22):15031–40. doi: 10.1039/d3ra01096a (PMC10186991; doi:10.1039/d3ra01096a)
Supplement: RA-013-D3RA01096A-s001 [file RA-013-D3RA01096A-s001.pdf]

Electronic Supplementary Material (ESI) for RSC Advances.
This journal is © The Royal Society of Chemistry 2023

Constructing Interface Engineering and Tailoring Nanoflower-like FeP/CoP Heterostructure Enhance Oxygen Evolution Reaction

Linhua Wang[a], Hua Yang[a]*, Lulan Wang[a], Yunwu Li[a], Wenning Yang[a], Xu Sun[b]*, Lingfeng Gao[b], Mingyu Dou[a], Dacheng Li[a], Jianmin Dou[a]*

[a] Shandong Provincial Key Laboratory of Chemical Energy Storage and Novel Cell Technology, School of Chemistry and Chemical Engineering, Liaocheng University, 252059 Liaocheng, P. R. China.

[b] Key Laboratory of Interfacial Reaction & Sensing Analysis in Universities of Shandong, School of Chemistry and Chemical Engineering, University of Jinan, Jinan 250022 Shandong, P. R. China.

E-mail: yanghua@lcu.edu.cn (H. Yang), dougroup@163.com (J.M. Dou).

Contents

Figure S1. XRD patterns of (a) CoP-350 and FeP-350; (b) samples with different input ratios; (c) samples with different phosphating temperatures.

Figure S2. SEM images of (a) FeP/CoP-0.5-350; (b) FeP/CoP-2-350; (c) CoP-350; (d) FeP-350; (e) FeP/CoP-1-300; (e) FeP/CoP-1-400 and (e) FeP/CoP-1-500.

Figure S3. EDS spectrums of (a) FeP/CoP-0.5-350; (b) FeP/CoP-2-350; (c) CoP-350; (d) FeP-350; (e) FeP/CoP-1-300; (e) FeP/CoP-1-400 and (e) FeP/CoP-1-500.

Figure S4. (a) Survey XPS spectra (b) C 1s high-resolution XPS spectra of FeP/CoP-1-350, CoP-350 and FeP-350.

Figure S5. LSV curve of (a) FeP/CoP-1-350, Carbon powder and Bare GCE; (b) FeP/CoP-1-350/CC and Bare CC.

Figure S6. CVs measurement with various scan rates for all samples with various input ratios and phosphating temperatures in 1 M KOH.

Figure S7. LSV curves normalized by ECSA.

Table S1. The values of charge transfer resistance (R_{ct}) and resistance (R_s) for different samples.

Table S2. The values of ECSA for different samples.

Table S3. The comparison of OER performance between this work and other reported transition metal phosphide catalysts.

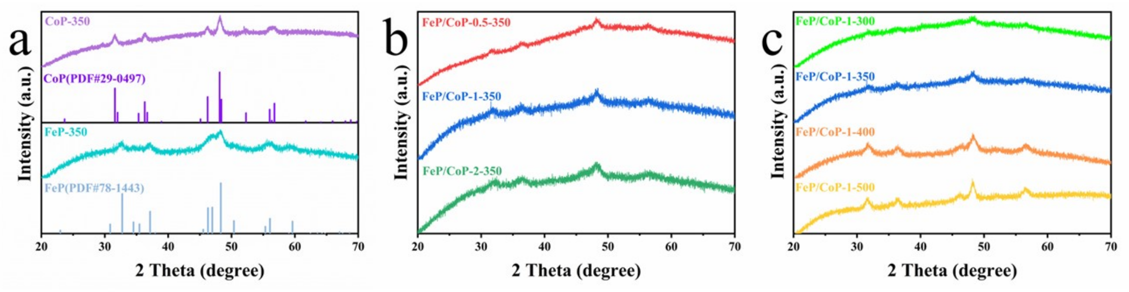

Figure S1. XRD patterns of (a) CoP-350 and FeP-350; (b) samples with different input ratios; (c) samples with different phosphating temperatures.

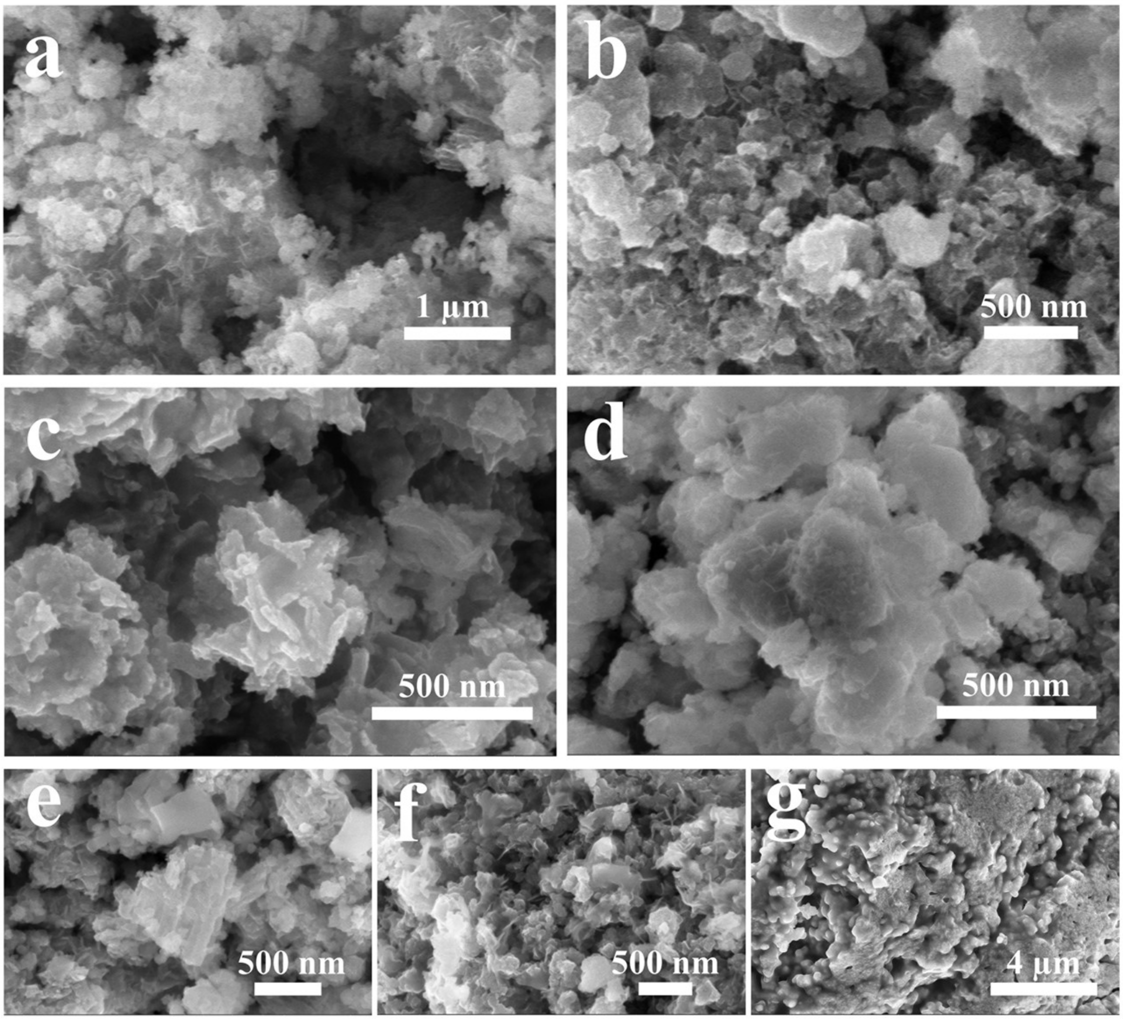

Figure S2. SEM images of (a) FeP/CoP-0.5-350; (b) FeP/CoP-2-350; (c) CoP-350; (d) FeP-350; (e) FeP/CoP-1-300; (f) FeP/CoP-1-400 and (g) FeP/CoP-1-500.

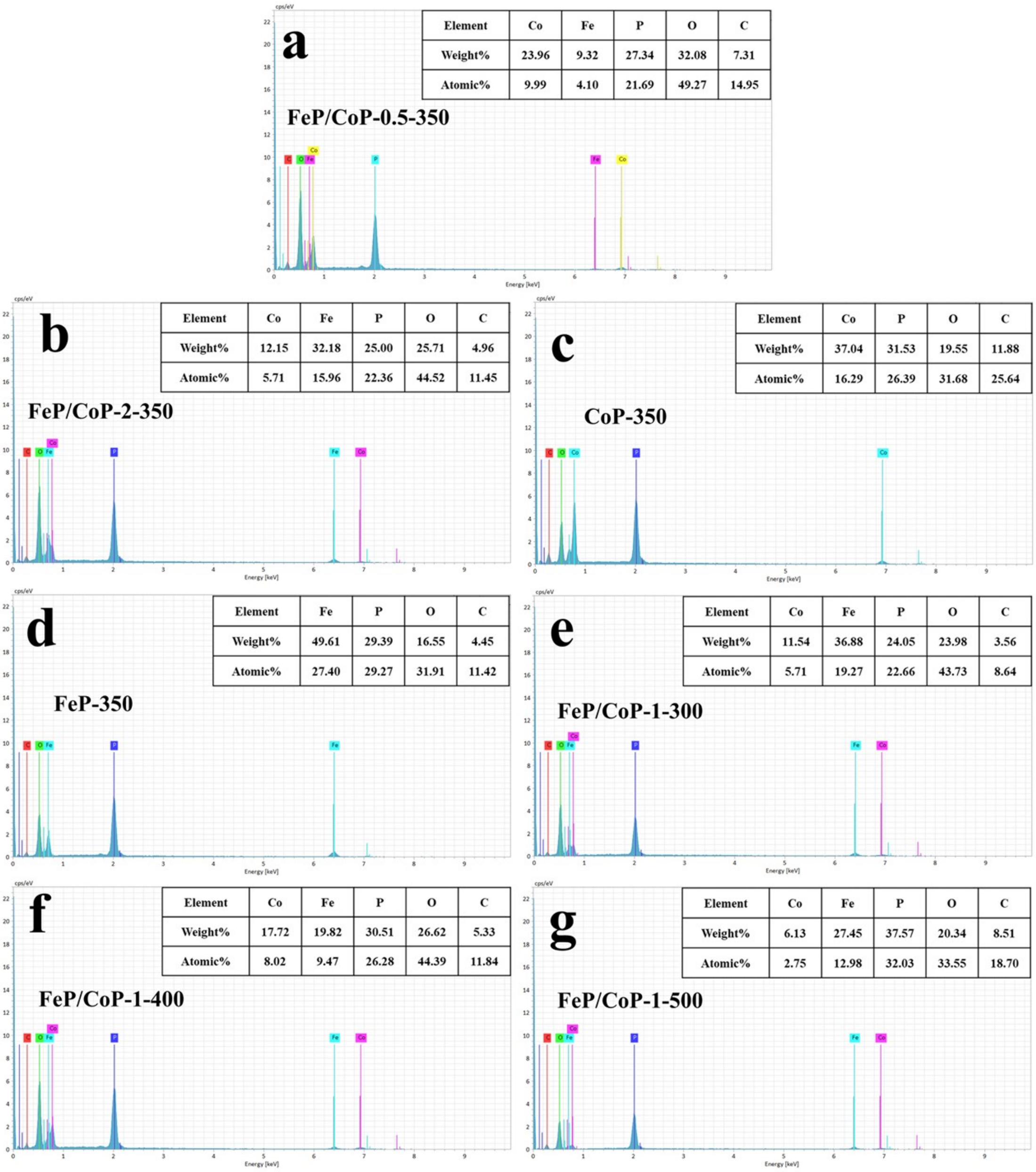

(a)

Element	Co	Fe	P	O	C
Weight%	23.96	9.32	27.34	32.08	7.31
Atomic%	9.99	4.10	21.69	49.27	14.95

(b)

Element	Co	Fe	P	O	C
Weight%	12.15	32.18	25.00	25.71	4.96
Atomic%	5.71	15.96	22.36	44.52	11.45

(c)

Element	Co	P	O	C
Weight%	37.04	31.53	19.55	11.88
Atomic%	16.29	26.39	31.68	25.64

(d)

Element	Fe	P	O	C
Weight%	49.61	29.39	16.55	4.45
Atomic%	27.40	29.27	31.91	11.42

(e)

Element	Co	Fe	P	O	C
Weight%	11.54	36.88	24.05	23.98	3.56
Atomic%	5.71	19.27	22.66	43.73	8.64

(f)

Element	Co	Fe	P	O	C
Weight%	17.72	19.82	30.51	26.62	5.33
Atomic%	8.02	9.47	26.28	44.39	11.84

(g)

Element	Co	Fe	P	O	C
Weight%	6.13	27.45	37.57	20.34	8.51
Atomic%	2.75	12.98	32.03	33.55	18.70

Figure S3. EDS spectrums of (a) FeP/CoP-0.5-350; (b) FeP/CoP-2-350; (c) CoP-350; (d) FeP-350; (e) FeP/CoP-1-300; (e) FeP/CoP-1-400 and (e) FeP/CoP-1-500.

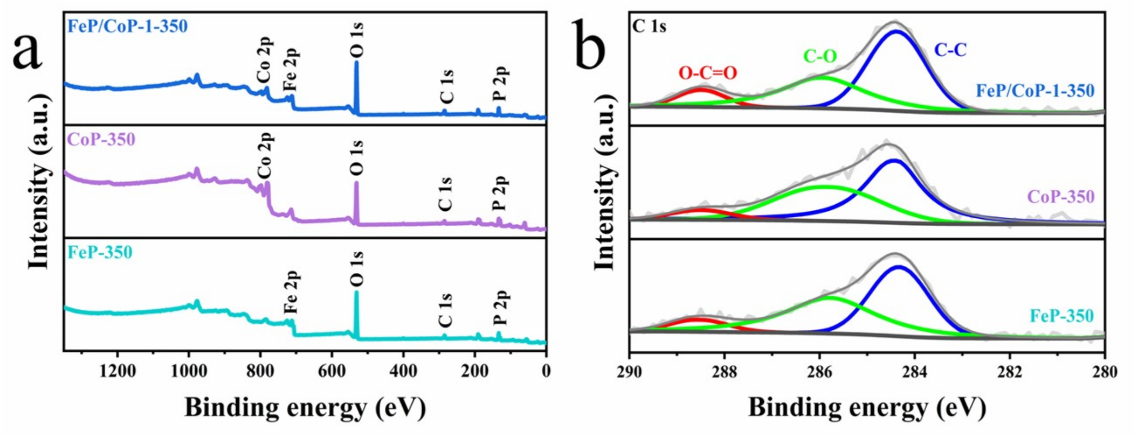

Figure S4. (a) Survey XPS spectra (b) C 1s high-resolution XPS spectra of FeP/CoP-1-350, CoP-350 and FeP-350.

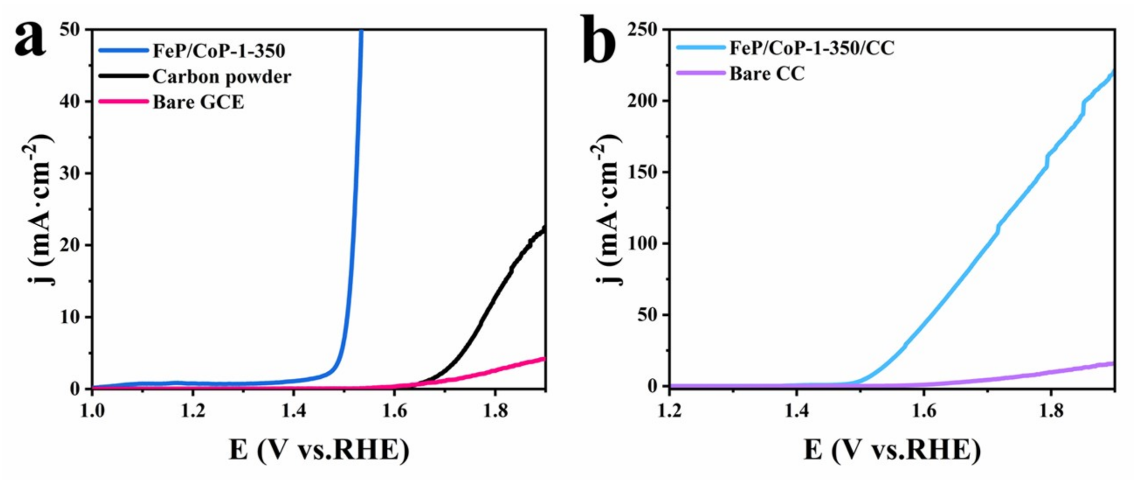

Figure S5. LSV curve of (a) FeP/CoP-1-350, Carbon powder and Bare GCE; (b) FeP/CoP-1-350/CC and Bare CC.

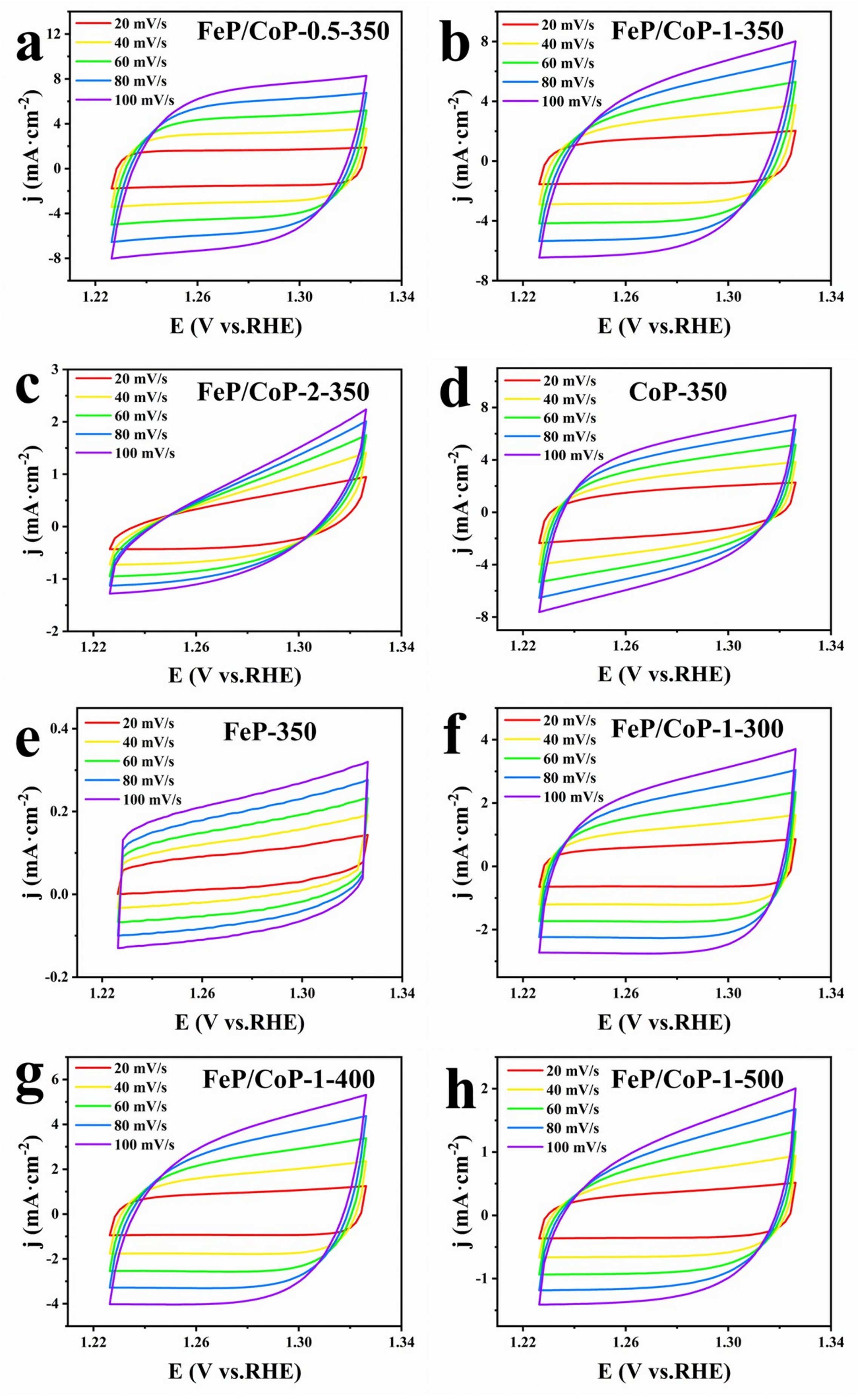

Figure S6. CVs measurement with various scan rates for all samples with various input ratios and phosphating temperatures in 1 M KOH.

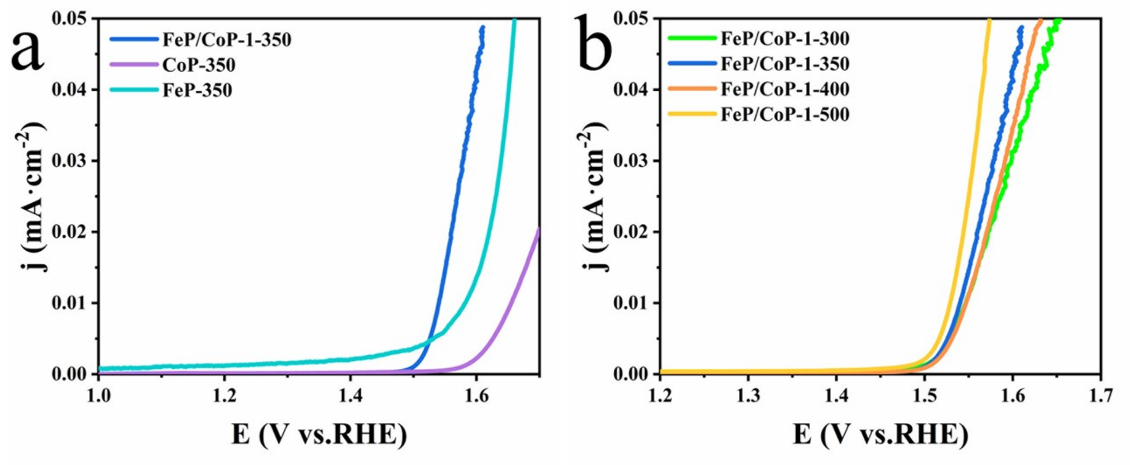

Figure S7. LSV curves normalized by ECSA.

Table S1. The values of charge transfer resistance (R_{ct}) and resistance (R_s) for different samples.

	R_s (Ω)	R_{ct} (Ω)
FeP/CoP-0.5-350	5.206	10.080
FeP/CoP-1-350	4.924	8.857
FeP/CoP-2-350	5.047	12.220
CoP-350	4.949	15.980
FeP-350	5.388	25.080
FeP/CoP-1-300	5.011	9.500
FeP/CoP-1-400	4.813	9.647
FeP/CoP-1-500	4.855	11.77

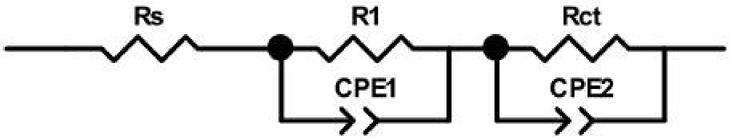

In the simplified equivalent electrical circuit, R_s is the overall series resistance, CPE_1 and R_1 are the constant phase element and resistance representing electron transport at CoFe-P catalyst/glassy carbon interface and between CoFe-P catalyst, respectively. CPE_2 is the constant phase element of the CoFe-P catalyst/electrolyte interface, and R_{ct} is the charge transfer resistance at CoFe-P catalyst/electrolyte interface related to the OER electrocatalysis process.

Table S2. The values of ECSA for different samples.

	ECSA (cm^2)
FeP/CoP-0.5-350	1709
FeP/CoP-1-350	1292.25
FeP/CoP-2-350	144
CoP-350	1084.5
FeP-350	38.25
FeP/CoP-1-300	642
FeP/CoP-1-400	896.75
FeP/CoP-1-500	286.25

Table S3. The comparison of OER performance between this work and other reported transition metal phosphide catalysts.

Electrocatalysts	Overpotential (mV)	Tafel slop ($mV\cdot dec^{-1}$)	Reference
FeP/CoP-1-350	276	37.71	This Work
NiCoFeP hollow nanoprism	294	50.5	S1
Cu-CoP nanosheets	411	101.4	S2
P@pCoPc/Co_3O_4 nanosheets	320	57.4	S3
CoP/NCNHP	310	70	S4
NiCoFeP films	300	124	S5
CoFeP	350	59	S6
$Ce_{0.5}$-CoP	365	96	S7
CoP/rGO	340	66	S8
CoP NFs	323	49.6	S9
Hollow Mo-CoP nanoboxes	305	56	S10
CoP-TiOx	337	72.1	S11
$Ni_{0.6}Co_{1.4}P$	300	80	S12

References

S1 B. He, C. Xu, Y. Tang, Y. Qian, H. Liu, Q. Hao and Z. Su, *J. Mater. Chem. A*, 2019, **7**, 24964-24972.

S2 L. Yan, B. Zhang, J. Zhu, Y. Li, P. Tsiakaras and P. Kang Shen, *Appl Catal B: Environ*, 2020, **265**, 118555.

S3 Y. Kim, D. Kim, J. Lee, L. Y. S. Lee and D. K. P. Ng, *Adv. Funct. Mater.*, 2021, **31**, 2103290.

S4 Y. Pan, K. Sun, S. Liu, X. Cao, K. Wu, W. C. Cheong, Z. Chen, Y. Wang, Y. Li, Y. Liu, D. Wang, Q. Peng, C. Chen and Y. Li, *J Am Chem Soc*, 2018, **140**, 2610-2618.

S5 S. Kan, M. Xu, W. Feng, Y. Wu, C. Du, X. Gao, Y. A. Wu and H. Liu, *ChemElectroChem*, 2021, **8**, 539-546.

S6 Y. Du, H. Qu, Y. Liu, Y. Han, L. Wang and B. Dong, *Appl. Surf. Sci.*, 2019, **465**, 816-823.

S7 J. Li, S. Zou, X. Liu, Y. Lu and D. Dong, *ACS Sustain Chem Eng*, 2020, **8**, 10009-10016.

S8 G. Zhang, B. Wang, J. Bi, D. Fang and S. Yang, *J. Mater. Chem. A*, 2019, **7**, 5769-5778.

S9 L. Ji, J. Wang, X. Teng, T. J. Meyer and Z. Chen, *ACS Catal*, 2019, **10**, 412-419.

S10 C. Guan, W. Xiao, H. Wu, X. Liu, W. Zang, H. Zhang, J. Ding, Y. P. Feng, S. J. Pennycook and J. Wang, *Nano Energy*, 2018, **48**, 73-80.

S11 Z. Liang, W. Zhou, S. Gao, R. Zhao, H. Zhang, Y. Tang, J. Cheng, T. Qiu, B. Zhu, C. Qu, W. Guo, Q. Wang and R. Zou, *Small*, 2020, **16**, 1905075.

S12 B. Qiu, L. Cai, Y. Wang, Z. Lin, Y. Zuo, M. Wang and Y. Chai, *Adv. Funct. Mater.*, 2018, **28**, 1706008.
